# Hydrodynamic Delivery of IL-10 Gene for Local Immunomodulation in Human Crohn’s Disease Tissue: A Proof-of-Concept Study

**DOI:** 10.3390/pharmaceutics18040442

**Published:** 2026-04-02

**Authors:** Luis Sendra, Francisco Giner, Gladys G. Olivera-Pasquini, María José Herrero, Enrique G. Zucchet, Salvador F. Aliño, Matteo Frasson

**Affiliations:** 1 Gene Therapy and Pharmacogenomics, Department of Pharmacology, Universitat de València, 46010 Valencia, Spain; luis.sendra@uv.es (L.S.); alino@uv.es (S.F.A.); 2 Pharmacogenetics and Gene Therapy Unit, Instituto de Investigación Sanitaria La Fe, 46026 Valencia, Spain; 3 Pathology Department, Hospital Universitario y Politécnico La Fe, 46026 Valencia, Spain; 4 Unit of Coloproctology Surgery, Division of General Surgery, Hospital Universitario y Politécnico La Fe, 46026 Valencia, Spain; 5 Digestive Surgery and Perioperative Care, Instituto de Investigación Sanitaria La Fe, 46026 Valencia, Spain

**Keywords:** IL-10, Crohn’s disease, gene therapy, hydrodynamic injection, arterial delivery, cytokine modulation, ex vivo human colon

## Abstract

**Background/Objectives**: Interleukin-10 (IL-10) is a potent anti-inflammatory cytokine that is critical for intestinal immune homeostasis. Despite its therapeutic potential, systemic delivery of IL-10 has failed in clinical trials for inflammatory bowel disease (IBD), largely due to its poor localization and short half-life. **Methods**: We present a proof-of-concept study demonstrating that hydrodynamic delivery of a naked plasmid bearing the human IL-10 gene to ex vivo human colonic segments from Crohn’s disease patients results in localized IL-10 expression and modulation of inflammatory mediators. **Results**: Compared to venous administration, arterial delivery yielded significantly higher IL-10 mRNA and protein levels, as well as decreased IL-6 and TNF-α expression. Furthermore, nanoparticle tracing confirmed efficient tissue penetration via the arterial route. **Conclusions**: These findings establish arterial hydrodynamic delivery as a feasible, non-viral strategy for targeted gene therapy in IBD.

## 1. Introduction

Crohn’s disease (CD) is a chronic, relapsing, and progressive inflammatory disorder of the gastrointestinal tract and constitutes one of the main clinical entities within inflammatory bowel disease (IBD). The disease is characterized by transmural inflammation, a discontinuous or “skip lesion” pattern, and the potential involvement of any segment of the gastrointestinal tract, although the terminal ileum and colon are most frequently affected [1]. Chronicity is primarily defined by the subsequent histoarchitectural and cytological alterations of glands secondary to epithelial injury following a prolonged and repetitive active and chronic inflammation, and its main characteristics are cryptal architectural alteration/distortion [2]. Histopathologically, CD is associated with crypt abscesses, granuloma formation, lymphoid aggregates, and extensive infiltration of innate and adaptive immune cells across all layers of the bowel wall [3]. These pathological changes cause the clinical manifestations and long-term complications of the disease.

Clinically, CD presents with abdominal pain, chronic diarrhea, weight loss, anemia, and fatigue, and may evolve toward complications such as strictures, fistulas, abscesses, and intestinal obstruction. Disease can progress over time from a mild inflammatory phenotype to stricturing or penetrating forms; these are associated with intestinal obstructions, which increase the likelihood of surgical intervention, and increased morbidity [4]. Despite improvements in disease control and treatment, CD remains a lifelong condition with a highly variable clinical course and significant impact on quality of life.

Over the last two decades, the therapeutic landscape of CD has widened substantially with the introduction of myriad biologic agents and small molecules targeting specific immune pathways that are altered in IBD [5]. Therapies directed against tumor necrosis factor alpha (TNF-α), integrins, and interleukin-12/23 signaling have improved outcomes for many patients. Nevertheless, a substantial proportion of individuals fail to achieve sustained remission [6]. Primary non-response rates remain clinically significant, and secondary loss of response frequently occurs due to immunogenicity, pharmacokinetic variability, or downstream pathway redundancy [7]. Furthermore, long-term systemic immunosuppression is associated with increased risks of infection, malignancy, and other immune-mediated adverse effects.

Even among patients who initially respond to advanced therapies, complete mucosal healing is not achieved, and disease progression may continue subclinically. Consequently, up to 70% of patients with CD ultimately require surgical bowel resection during the course of their disease. Furthermore, surgery is not completely curative and postoperative recurrence is common, highlighting the need for novel therapeutic strategies that effectively address the immune dysregulation while minimizing systemic exposure [8].

At the immunological level, CD results from an inappropriate and sustained immune response to luminal antigens. This response mediates a complex interplay between host genetics, the intestinal microbiota, epithelial barrier function, and dysregulated innate and adaptive immune signaling [9]. Genome-wide association studies have identified susceptibility loci involved in microbial sensing, autophagy, epithelial integrity, and immune regulation [10]. In parallel, alterations in microbial composition and function contribute to aberrant immune activation [11].

Pro-inflammatory cytokines produced in response to pathogens such as IL-1β, TNF-α, and IL-17 regulate antimicrobial peptide production, increase epithelial permeability, and drive immune cell recruitment [12]. M1 macrophages, induced by interferon-gamma (IFN-γ) and lipopolysaccharide (LPS), are characterized by their pro-inflammatory properties. They produce high levels of cytokines such as tumor necrosis factor-alpha (TNF-α), interleukin-1 beta (IL-1β), interleukin-6 (IL-6), interleukin-12 (IL-12), and interleukin-23 (IL-23), which are crucial for initiating and sustaining inflammatory responses [13]. Counter-regulatory mechanisms that normally balance these responses in healthy people are insufficient or defective in CD patients, allowing inflammation to persist despite endogenous anti-inflammatory signals. Among endogenous anti-inflammatory mediators, interleukin-10 (IL-10) plays a pivotal and non-redundant role in maintaining intestinal immune homeostasis [14]. IL-10 is a pleiotropic cytokine produced by regulatory T cells, macrophages, dendritic cells, B cells, and intestinal epithelial cells [15]. It acts primarily by inhibiting antigen presentation, suppressing pro-inflammatory cytokine production, and limiting effector T-cell activation, while simultaneously promoting immune tolerance to commensal microbiota. Within the intestinal mucosa, IL-10 signaling is essential to prevent excessive immune activation in response to continuous antigenic exposure [16]. IL-10 plays a non-redundant role in suppressing mucosal inflammation, and genetic deficiencies in IL10 pathway can cause severe IBD phenotypes [17,18,19].

The critical importance of IL-10 is supported by genetic and experimental evidence. IL-10-deficient mice develop spontaneous, microbiota-driven colitis characterized by uncontrolled Th1 and Th17 immune responses [20]. In humans, loss-of-function mutations in IL10 or its receptor subunits (IL10RA and IL10RB) result in severe, early-onset enterocolitis that is frequently refractory to immunosuppressive and biologic therapies [21]. These observations clearly establish IL-10 signaling as necessary for intestinal immune regulation and point out IL-10 as an attractive therapeutic target in IBD. Based on this strong biological rationale, recombinant IL-10 was evaluated in clinical trials for CD. However, these studies failed to demonstrate consistent clinical benefit, and dose escalation was limited by systemic adverse effects [22,23]. Several explanations have been proposed for this lack of efficacy, including rapid systemic degradation, short plasma half-life, inadequate delivery to inflamed intestinal tissue, and the inability to maintain therapeutically relevant local concentrations. It must be underlined that systemic administration does not replicate the local paracrine action of endogenous IL-10 within the intestinal mucosa [24].

These limitations have renewed interest in alternative strategies for restoring IL-10 activity directly at the site of inflammation. Gene therapy approaches can potentially mediate sustained, localized production of therapeutic proteins within target tissue, overcoming the pharmacokinetic and biodistribution challenges associated with recombinant cytokines. Viral vectors have demonstrated efficacy in preclinical models of colitis; however, concerns regarding immunogenicity, safety, cargo capacity, and restricted repeat dosing have limited their clinical translation. Non-viral gene delivery methods represent a safer and more flexible strategy. Among these, hydrodynamic gene transfer has emerged as a powerful physical technique capable of delivering naked plasmid DNA into cells by transiently increasing intravascular pressure. This process induces reversible permeabilization of endothelial and cellular membranes, allowing DNA to access the cytoplasm and nucleus without chemical or viral carriers. Initially developed for liver-directed gene transfer in rodents, hydrodynamic injection has demonstrated high transfection efficiency and robust transgene expression with a favorable safety profile [25,26]. More recently, adaptations of hydrodynamic gene transfer have been explored in large-animal models [27,28,29,30]. In previous works of our group, isolated human organs like liver [31] and colon [32] were also employed, with promising results, suggesting its potential translational applicability. However, its feasibility in human intestinal tissue affected by chronic inflammatory disease has not been previously investigated. In particular, CD-associated vascular remodeling, fibrosis, and mucosal damage could limit its catheter-mediated access and the effective gene delivery.

Hydrodynamic gene transfer is an alternative strategy to enable localized IL-10 expression in inflamed intestinal tissue. This procedure facilitates plasmid DNA uptake through rapid intravascular injection, which has been previously demonstrated in hepatic gene transfer models [27,32]. In this proof-of-concept study, we therefore investigate the application of hydrodynamic gene transfer for localized delivery of a plasmid encoding human IL-10 to “ex vivo” colonic specimens from CD patients, comparing arterial versus venous routes to evaluate delivery efficiency, distribution, transgene expression, and functional cytokine modulation.

## 2. Materials and Methods

### 2.1. Human Tissue and Delivery Protocol

Colon segments (*n* = 12) were obtained from CD patients undergoing laparoscopic ileocecal resection as follows. The Declaration of Helsinki protocols were followed, and patients gave their written informed consent. This research was approved by the clinical research ethics Committee of the University Hospital La Fe, Valencia (Spain). A standard multiport technique was used, including an umbilical camera port and two to three working trocars. The terminal ileum and right colon were mobilized with preservation of retroperitoneal structures. Once the diseased segment had been identified, a small periumbilical incision was created, allowing exteriorization of the bowel. The ileocolic vessels were identified and ligated to ensure vascular control. Resection of the diseased ileocecal segment from twelve CD patients, including 10–20 cm of terminal ileum and the cecum, was then completed extracorporeally. Restoration of bowel continuity was achieved through an extracorporeal ileocolic anastomosis. We used 2 samples for the procedure feasibility assessment (Control). One tissue segment was used to evaluate the gold nanoparticles’ distribution. We could finally transfer the IL10 gene in 9 colonic segments. Six of these were transferred following a venous route only, two were transferred by both venous and arterial routes, and one was transferred through an arterial route only. This was due to the difficulty of arterial cannulation because of the strong vascular resistance and vessel retraction “ex vivo”. Control tissue samples were obtained from distal areas of each segment that were previously vascularly excluded with a clamp; therefore, 11 control samples were available.

The plasmid p2F-hIL10 (6.86 Kb), containing the human IL10 protein cDNA (intronless ORF from the ATG to the stop codon, size: 537 bp) was purchased from Invivogen based on pVITRO2 (Invivogen, Toulouse, France) backbone and driven by a ferritin FerH (heavy-chain) promoter, which was constructed by cloning hIL10 into the Hind III site. This plasmid was administered (*n* = 11) using hydrodynamic injection (20 µg/mL, 2 mL/cm tissue) via either arterial or venous routes after radiologic confirmation of the irrigated area at rate of 5–7 mL/s.

### 2.2. Tissue Culture

Tissue samples representing the whole colon wall were divided into two groups: control and treated (artery and vein). The control samples were those obtained from the ends of the segments in regions without perfusion due to vascular exclusion mediated by clamps and confirmed by X-rays. All samples were cut into small pieces (2 × 1 cm^2^) and cultured in DMEM medium at 37 °C and 5% CO_2_ atmosphere. Samples were collected for further analyses 1, 3, 5 and 7 days after gene transfection. Culture medium was completely renewed every time a sample was collected to maintain culture conditions. For this reason, it is necessary to consider the amount of every protein detected in the supernatant that was produced in the period between sample collections (24 or 48 h).

### 2.3. Gene and Protein Analysis

Quantification of IL10 mRNA could only be performed on samples that were not degraded (control *n*: 4; vein-treated *n*: 4; artery-treated *n*: 3). Quantitative RT-PCR was performed at defined time points (0, 1, 3, 5 and 7 days) to assess hIL10 mRNA levels in tissue after its extraction and purification. For quantitative real-time qPCR, TaqMan PCR master mix (Thermofisher, Watham, MS, USA) was employed according to the instructions of the manufacturer. The specific oligonucleotides for human IL-10 employed were from a pre-mixed TaqMan kit from Life Technologies (Carlsbad, CA, USA), cat no. Hs00961622_m. Quantitative data were calculated as the number of RNA copies on a regression curve; the curve was plotted with the injected plasmid containing the hIL10 gene. The curve was prepared with a known concentration of hIL10 plasmid and serial 1/10 dilutions. The linearity of the standard curve included from 10^3^ to 10^7^ copies with a correlation coefficient > 0.95. The data were expressed as copies per cell considering the average content of RNA per cell in a mammalian cell (20 pg) for gene transcription.

For human IL10 protein detection, we collected culture supernatants. The BD OptEIA® Human IL10 ELISA Set commercial kit (Beckton and Dickinson Biosciences, Franklin Lakes, NJ, USA) was used according to the manufacturer’s instructions. We ultimately included samples from 7 colonic segments of the control group, 8 from venous-treated samples and 3 from artery-treated segments. The standard curve was prepared using purified hIL10 protein. In our hands, it was a lineal standard curve from 7.8 pg/mL to 500 pg/mL with a correlation coefficient > 0.99. A multiplex immunoassay (Cytokine 25-Plex Human Panel; Invitrogen, ThermoFisher scientific, Waltham, MA, USA) profiled changes in 25 cytokines, focusing on IL-6, TNF-α, IL-2R, and IFN-α. Since no significant difference was observed between the venous and arterial route regarding IL10 expression, the results were represented together; therefore, for these results, control *n*: 10 and treated *n*: 10.

### 2.4. Nanoparticle Preparation and Tracking by Transmission Electron Microscopy

All chemicals were purchased from Sigma-Aldrich (St Louis, MO, USA) and used as received, without further purification. Gold nanoparticles were synthesized following a modification of the Turkevich method, as previously described [32]. A human colon segment (*n* = 1) was hydrodynamically injected (2 mL/cm) through the catheter with a buffered solution of 2.5% glutaraldehyde containing 10^12^ gold particles per ml to study the colon distribution of 4 and 15 nm gold nanoparticles after injection. Then, small tissue pieces from different colon areas were removed and immersed in phosphate Sørensen buffer (pH 7.4) solution containing 2.5% glutaraldehyde. For TEM, multiple 1 mm^3^ pieces of colon wall were routinely processed and embedded in Epoxy resin. Ultrathin sections stained with uranyl acetate were examined under a Jeol JEM-1010 electron microscope (Jeol ltd, Tokyo, Japan). It must be underlined that, to better identify the gold nanoparticles in tissue, sections were not stained with lead, as usual. Although this reduces the definition of tissue anatomical features, it facilitates the observation of nanoparticles and circumvents the low contrast that they present within the tissue.

### 2.5. Statistical Analyses

When three groups were compared, a two-way ANOVA was performed, taking into account the repeated-measures nature of the data. In cases where missing values were present, a mixed-effects model was used. For comparisons between two groups, a paired *t*-test was applied. Statistical significance was defined as *p* < 0.05. The results graphing and statistical analyses were conducted using Prism version 10 software (GraphPad Software, Boston, MA, USA).

## 3. Results

### 3.1. Evaluation of Vascular Accessibility

**Figure 1:**
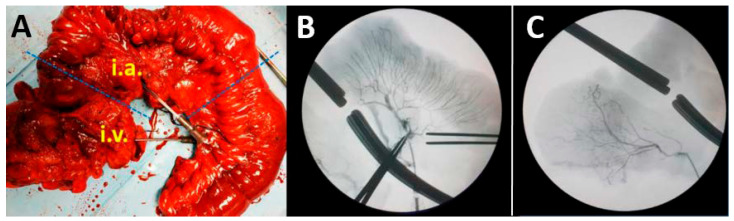
Example of arterial and venous vascularization of a colon segment affected by Crohn’s disease. The left panel shows a Crohn’s disease-affected colonic segment with catheters placed for gene injection (**A**). The middle and right panel are radiologic X-ray images showing tributary areas of a colonic artery (**B**) and a vein (**C**), respectively. Iodinated contrast solution was employed for its visualization. i.a.: intraarterial; i.v.: intravenous.

Permeation of tissue through selected vessels was verified in every experiment to assess the correct irrigation of the segment (Figure 1) and to define the tissue tributary of the vessel employed.

### 3.2. Tissue and Cell Access of Gold Nanoparticles

**Figure 2:**
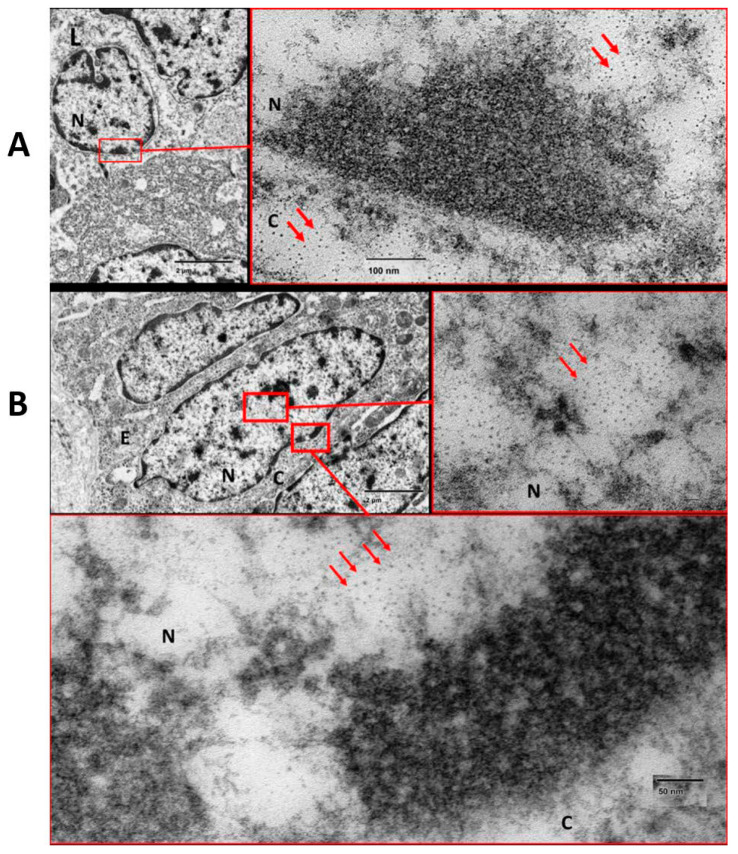
TEM images of gold nanoparticle (4 nm diameter) distribution in mucosa of Crohn’s-affected colonic tissue. (**A**) shows a lymphocyte. (**B**) shows a transverse cut of the enterocytes. Red arrows mark the presence of gold nanoparticles. C: cytoplasm; N: nucleus; E: enterocyte; L: lymphocyte.

The distribution of 4 and 15 nm diameter gold nanoparticles after hydrodynamic injection was studied in mucosal layer of colonic tissue (*n* = 1) by transmission electron microscopy. TEM revealed that 4 nm gold nanoparticles, but not 15 nm particles, penetrated deeply into the mucosal, submucosal, and muscular layers (Figure 2). Particles were observed in cell nuclei following injection. The upper panel (A) shows the presence of a lymphocyte. The red squared area of the nuclear envelope is enlarged in the right image and nanoparticles can be observed at both sides. The left image of the middle panel (B) shows a transverse cut of the enterocytes. Red squared areas were enlarged in the right image, which shows gold nanoparticles inside the nucleus that are preferentially distributed near nuclear pores. The lower panel is an augmented image of the enterocyte nuclear envelope.

### 3.3. IL-10 Transgene Expression

**Figure 3:**
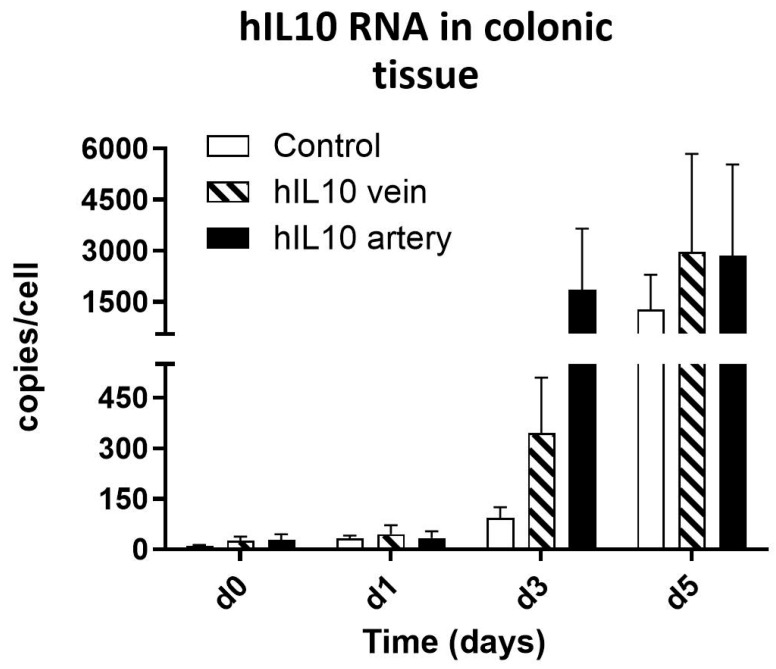
Human IL10 mRNA expression in colonic tissue samples expressed as copies/cell.

Once we had confirmed the feasibility of vascular irrigation and the wide distribution of gold nanoparticles with similar sizes to the plasmid administered, the expression of the transferred gene was evaluated (*n* = 11). Tissue samples were homogenized and RNA was extracted and purified. RT-qPCR revealed robust IL-10 transcription (Figure 3) in treated samples, peaking at day 5 with levels exceeding 2900 RNA copies/cell—approximately 10-fold higher than the observed in control samples. Arterial injection yielded ~2.5-fold higher expression than venous delivery on day 3. Control tissue showed a more modest increase (max 1200 copies/cell), possibly due to the inflammatory status. Day 7 results were not represented because the tissue was degraded.

### 3.4. Interleukin-10 Protein Production

**Figure 4:**
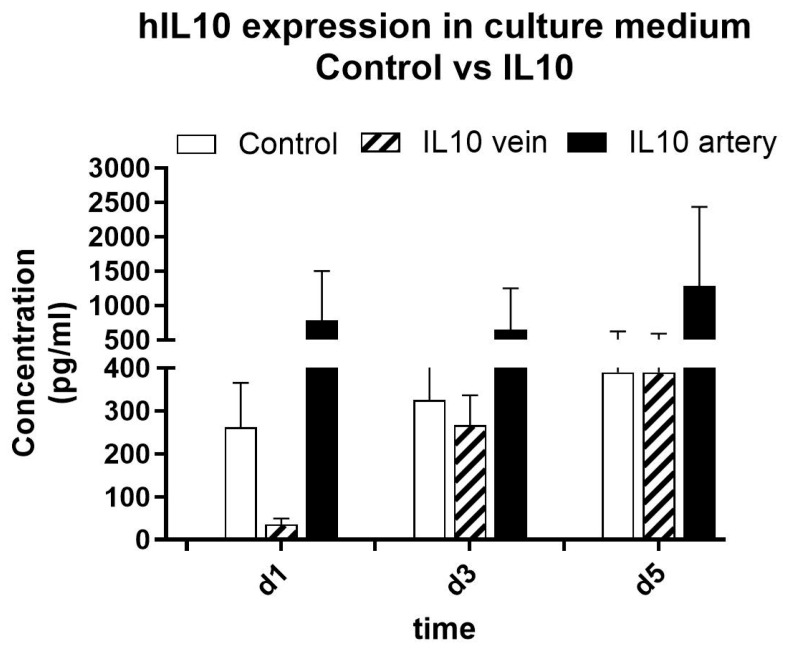
Human IL10 protein concentration in culture supernatant of colonic tissue samples. Culture medium was renewed completely at every sampling collection time.

To verify the completion of the decoding process, IL-10 protein was quantified in culture medium. DMEM was collected on day 1, day 3 and day 5 to quantify the concentration of human IL-10 that had been translated and released by tissue samples and accumulated for 24 h (d1) or 48 h (d3 and d5). Although results were dispersed, ELISA confirmed functional protein secretion (Figure 4), with arterial delivery achieving over 1200 pg/mL by day 5, vs. <400 pg/mL in venous or control samples. Protein production declined by day 7 due to tissue degradation. The levels of IL10 protein observed in venous-treated samples were lower than in control on day 1. We believe that the reason for the decrease in protein concentration could be due to the first washing effect mediated by the gene solution injection. We do not know the exact reason why we did not observe that result when we used the arterial route of administration, but we guessed that this could be due to the limited flow rate in arterial route or the higher efficacy in gene transfer when employing it.

### 3.5. Effect on Inflammatory Cytokines

**Figure 5:**
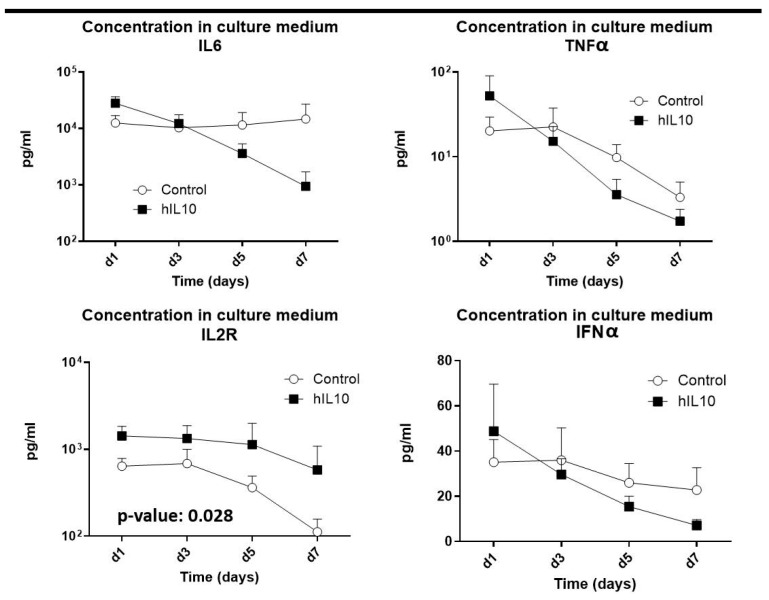
Concentration of IL-6, TNFα, IL-2R and IFNα cytokines in culture supernatant of colonic tissue samples was quantified. Culture medium was renewed completely at every sampling collection time.

After confirming that IL-10 was translated and released, it was important to find out whether the overexpression induced a functional effect on immunomodulation. Multiplex analyses (Figure 5) were performed on culture medium and the results indicated a progressive reduction in IL-6 and TNF-α levels in treated samples as compared to controls (up to 14-fold lower IL-6 on day 7, though this reduction was not statistically significant). These results show an anti-inflammatory trend in samples treated with hIL-10 gene. Soluble IL-2 receptor levels were significantly elevated (*p* = 0.028), suggesting that this may modulate IL-2 signaling by sequestering interleukin-2 molecules and preventing its binding to membrane IL-2R, thus reducing the effector response. IFN-α levels also declined post-treatment. All these results together suggest modulation of the inflammatory status in treated samples.

## 4. Discussion

The present study provides proof-of-concept evidence that hydrodynamic arterial gene transfer enables localized, biologically active IL-10 expression in human Crohn’s disease colonic tissue. This finding is particularly relevant given the well-recognized limitations of systemic cytokine therapy and the complex vascular remodeling, fibrosis, and architectural distortion that characterize chronic intestinal inflammation in Crohn’s disease patients [1,2,3]. Despite the central anti-inflammatory role of interleukin-10 [14], systemic administration has historically shown limited therapeutic efficacy, largely due to its short half-life, dose-limiting adverse effects, and insufficient mucosal bioavailability [22,23]. Clinical trials of recombinant IL-10 in inflammatory bowel disease highlighted these pharmacokinetic and safety constraints, underscoring the need for localized delivery strategies capable of achieving therapeutic concentrations at the site of inflammation while minimizing systemic exposure.

The superior efficiency observed with arterial delivery likely reflects more favorable intravascular pressure gradients and a more homogeneous distribution within the submucosal and mucosal vascular plexus. Chronic inflammatory tissue in Crohn’s disease exhibits increased vascular permeability, angiogenesis, and altered microcirculatory flow [33], factors that may enhance transvascular plasmid passage during transient hydrodynamic pressure elevations. These mechanistic considerations are consistent with prior preclinical work demonstrating that hydrodynamic gene transfer can achieve high levels of transgene expression in solid organs such as the liver [27], and extend those observations to structurally complex, chronically inflamed human intestinal tissue. The ultrastructural evidence demonstrating nuclear localization of small nanoparticles strengthens this interpretation, as successful plasmid-based gene expression requires cytoplasmic trafficking and nuclear entry. The visualization of nuclear compartment access supports the biological plausibility of effective transcriptional activation within epithelial and lamina propria cells.

Importantly, IL-10 overexpression resulted in functional suppression of key pro-inflammatory mediators, including interleukin-6 and tumor necrosis factor alpha, both of which play central pathogenic roles in Crohn’s disease [13]. The pathogenic relevance of TNF-α is underscored by the clinical success of anti-TNF biologics, which remain a mainstay of therapy [34]. The observed downregulation of IL-6 and TNF-α in the present study indicates that the expressed IL-10 was not merely detectable, but biologically active within the inflammatory microenvironment. Moreover, the increase in soluble IL-2 receptor levels suggests modulation of T-cell activation dynamics. Given IL-10’s established capacity to inhibit antigen-presenting cell function, reduce Th1 and Th17 polarization, and promote regulatory T-cell responses, these findings align with known immunoregulatory pathways [15,16] and suggest that localized gene delivery can recapitulate physiologically relevant anti-inflammatory circuits. Although the concentrations were different, we observed that the control and treated samples yielded a similar trend in cytokines expression. We do not know the exact reason for this, but we cannot exclude that this could be due to the “ex vivo” conditions of our experiments.

These results extend prior animal models of IL-10 gene therapy and earlier “ex vivo” human tissue studies reported by our group [27,32] by specifically addressing chronically inflamed Crohn’s disease specimens, which more closely reflect the therapeutic challenges encountered in clinical practice. Non-viral gene therapy approaches are particularly attractive in this context due to their favorable safety profile compared with viral vectors [25,26], including reduced risks of insertional mutagenesis and vector-specific immune responses. While viral systems have demonstrated higher transfection efficiency in some settings, safety concerns [35] have limited their translational momentum in non-life-threatening chronic diseases such as inflammatory bowel disease.

The “ex vivo” nature of the model limits conclusions regarding systemic immune crosstalk, durability of expression, vector re-administration feasibility, and long-term safety. It does not capture dynamic interactions among circulating immune cells, the microbiota, and mesenteric lymphoid compartments, all of which contribute to disease perpetuation. Nevertheless, the model provides a highly controlled and disease-relevant human platform to evaluate transfection feasibility, tissue penetration, and short-term biological activity under conditions that preserve native architecture and inflammatory signaling networks. As such, it represents a critical translational bridge between preclinical experimentation and potential “in vivo” application.

## 5. Conclusions

A hydrodynamic injection procedure is an efficient model for the delivery of naked DNA to cell nuclei of human Crohn’s disease colon segments. It permits the efficacious expression of the protein encoded by the transferred gene. In this work, functional anti-inflammatory responses of human IL10 could be observed, supporting its potential as a gene therapy platform for IBD and other pathologies employing any nucleic acid molecule, with a diameter of over 15 nm being a limitation. The fact that arterial injection yielded higher efficacy could be of interest for its clinical application, since this route is more easily accessible via catheter-mediated procedures. Taken together, these findings support the concept that hydrodynamic arterial gene transfer may overcome key barriers that have historically limited cytokine-based therapies in Crohn’s disease. By enabling localized, biologically active IL-10 expression within diseased intestinal tissue, this strategy offers a mechanistically rational and potentially safer alternative to systemic cytokine administration, warranting further investigation in in vivo translational models and, ultimately, early-phase clinical studies.

## Data Availability

The original contributions presented in this study are included in the article. Further inquiries can be directed to the corresponding author.

## Abbreviations

The following abbreviations are used in this manuscript:

ATG	Adenine–Thymine–Guanine—the universal start codon.
CD	Crohn’s disease.
cDNA	Complementary DNA.
DMEM	Dulbecco’s Modified Eagle’s Medium.
DNA	Desoxyribonucleic acid.
ELISA	Enzyme-Linked Immunosorbent Assay.
FerH	Heavy chain of ferritin.
IBD	Inflammatory bowel disease.
IFN	Interferon.
IL	Interleukin.
mRNA	Messenger ribonucleic acid.
RNA	Ribonucleic acid.
RT-PC	Reverse Transcription Polymerase Chain Reaction.
qPCR	Quantitative Polymerase Chain Reaction.
TEM	Transmission Electron Microscopy.
TNF	Tumor necrosis factor.
